# Alcohol-induced accumbal dopamine- and taurine release in female and male Wistar rats, an in vivo microdialysis study

**DOI:** 10.1007/s00702-025-02928-w

**Published:** 2025-04-18

**Authors:** Anna Loftén, Karin Ademar, Klara Danielsson, Bo Söderpalm, Louise Adermark, Mia Ericson

**Affiliations:** 1 Addiction Biology Unit, Department of Psychiatry and Neurochemistry, Institute of Neuroscience and Physiology, The Sahlgrenska Academy at University of Gothenburg, Box 410, 405 30 Gothenburg, Sweden; 2 Beroendekliniken, Sahlgrenska University Hospital, Gothenburg, Sweden; 3 Department of Pharmacology, Institute of Neuroscience and Physiology, The Sahlgrenska Academy at University of Gothenburg, Gothenburg, Sweden

**Keywords:** Alcohol, Dopamine, Nucleus accumbens, Rat, Sex differences, Taurine

## Abstract

Alcohol use disorder (AUD) is a relapsing brain disorder involving major neurobiological changes. Upon alcohol exposure, dopamine (DA) levels increase in the nucleus accumbens (nAc), a key region of the mesolimbic DA system involved in reward and reinforcement. A concomitant increase in extracellular taurine within the nAc has been shown to be important for the alcohol-induced DA increase. Sex differences in alcohol consumption and in the development of AUD have previously been shown. However, knowledge regarding sex differences in alcohol-induced DA and concomitant taurine release is limited. The aim of this study was to examine potential sex differences in alcohol-induced increases of extracellular levels of DA and taurine within the nAc, following local and systemic alcohol administration. To this end, in vivo microdialysis was performed using male and female Wistar rats. Following systemic alcohol administration, both male and female rats displayed a significant increase of both DA and taurine within the nAc, with no observed sex differences. In contrast, males displayed a significant increase in both DA and taurine following alcohol administration locally into the nAc whilst female rats displayed a blunted DA response and an attenuated taurine increase. Basal levels of DA or taurine did not differ significantly between males and females. The results presented here suggest that local accumbal mechanisms contribute to a greater extent to the alcohol-induced DA increase in male compared to female rats, whilst the response to systemic alcohol administration is similar between sexes.

## Introduction

Alcohol use disorder (AUD) is a debilitating brain disorder causing great morbidity and mortality, and the affected individuals have significantly reduced life expectancy and suffer from severe medical consequences as a result of long-term alcohol use (Rehm and Shield 2019). Globally, men are overrepresented among those diagnosed with AUD, however, the number of women with problematic alcohol consumption is steadily increasing (Grant et al. 2017; Grucza et al. 2018; White et al. 2015). The progress of the disorder seems to be more severe in women than in men (Kirsch et al. 2025; Piazza et al. 1989; Randall et al. 1999). Additionally, females show higher comorbidity with other psychiatric and medical disorders than men (Erol and Karpyak 2015; Mann et al. 2004). Some of the differences between men and women and their susceptibility for drug consumption and consequences are suggested to be related to sex-specific mechanisms (McHugh et al. 2018). Females express lower levels of gastric alcohol dehydrogenase whilst showing a higher hepatic alcohol metabolism, leading to increased acetaldehyde-mediated toxicity within the liver and consequently alcohol-related liver disease. Due to different body composition with a relatively lower water content, women display lower distribution volume leading to higher systemic alcohol levels following alcohol consumption of the same quantity/kg (Bizzaro et al. 2023).

In rodents, consummatory behaviours differ between males and females. Female rodents are known to consume more alcohol (relative to body weight) than their male counterparts (Rath et al. 2020; Sneddon et al. 2019). Moreover, female rats have been shown to be more resistant to aversive additives in the alcohol drinking solution (Foo et al. 2023; Radke et al. 2020), and are more resistant to punishment during alcohol intake (McElroy et al. 2023), suggesting a higher resilience to negative consequences. Anatomical differences of reward-related brain areas have been described (Forlano and Woolley 2010), and sex hormones appear to further impact alcohol intake (Almeida et al. 1998; Venegas et al. 2024). Thus, the rewarding effects by alcohol may be highly sex-dependent and fluctuate according to hormonal cycles (Cao et al. 2018; Dazzi et al. 2007; Krentzel et al. 2021; Vandegrift et al. 2017; Venegas et al. 2024; Yoest et al. 2019).

While many signalling pathways are activated during alcohol exposure (Vengeliene et al. 2008), the mesolimbic dopamine (DA) system, comprising of dopaminergic neurons projecting from the ventral tegmental area (VTA) to the nucleus accumbens (nAc), plays a crucial role in reward processing (Di Chiara and Imperato 1988). Activation of this system leads to an increase in extracellular DA within the nAc, an event associated with reward (Koob 1992; Wise and Rompre 1989). The alcohol-induced increase in DA is mediated through various neurotransmitter systems via mechanisms not fully elucidated. Glycine receptors (GlyRs) (Söderpalm et al. 2017) and cholinergic interneurons (Loftén et al. 2021; Wadsworth et al. 2023) within the nAc seem to play a crucial role in the DA increase. Additionally, the alcohol-induced DA elevation in the nAc is accompanied by an increase in taurine levels (Adermark et al. 2011; Dahchour et al. 1996; Ericson et al. 2011). This increase in extracellular taurine is suggested to be pivotal for the alcohol-induced DA elevation, as inhibiting the taurine release results in attenuation of the DA increase following alcohol exposure (Ericson et al. 2011). Taurine itself can induce a dopamine increase in the nAc when applied locally, likely through its action on GlyRs (Ericson et al. 2006). Taurine is not only involved in osmoregulation, and neuroprotection, but also regulates calcium homeostasis and supports cognitive processes (Lambert et al. 2015). Its effects are mediated through interactions with neurotransmitter systems, particularly glycinergic and glutamatergic pathways where taurine is an agonist of GlyRs inducing responses at μM levels. Taurine also exerts a weak agonistic effect on GABA_A_ receptors where mM concentrations of taurine are typically needed to induce responses (Albrecht and Schousboe 2005; Jiang et al. 2004). Furthermore, taurine has been shown to directly interact with glutamatergic NMDA receptors (Chan et al. 2014), which demonstrates intricate actions on the brain. Adding to the complexity, the effects of taurine vary across different brain regions, and the baseline levels has been reported to be higher in males in certain areas of the brain (Tkáč et al. 2023).

Few studies have investigated sex differences in alcohol-induced elevation of accumbal DA, and it has only been done following systemic alcohol administration (Blanchard and Glick 1995). Studies investigating sex differences in the alcohol-induced taurine release are likewise scarce and the findings are contradictory (Ademar et al. 2024; Adermark et al. 2011; Ericson et al. 2011, 2020; Lallemand et al. 2011). The aim of this study was to investigate potential sex differences in the alcohol-induced increase of extracellular levels of DA and taurine within the nAc following acute local and systemic alcohol administration. To this end, in vivo microdialysis was performed using male and female Wistar rats. Additionally, the study aimed to determine the local tissue concentration of alcohol in the nAc, adjacent to the implanted microdialysis probe, following local and systemic alcohol administration.

## Method and materials

### Animals

Adult male and female Wistar rats (Envigo, Netherlands, n = 90), weighing 280–350 g, corresponding to the age of 9–10 weeks, at the time of the experimental procedure, were used. The animals were group housed until surgery, in a controlled environment with regular dark–light conditions (lights on at 7 AM and off at 7 PM) and ad libitum access to tap water and standard rat chow. The animals were allowed to acclimate to the environment for at least 5 days before surgery. The experiment was conducted in accordance with protocols approved by the Ethics Committee for Animal Experiments, Gothenburg, Sweden (2401/19).

### Drugs

Alcohol (ethanol (EtOH) 95% Kemetyl AB, Haninge, Sweden or 95% Kiilto Clean AB, Täby, Sweden) was diluted in Ringer’s solution (consisting of (in mmol/l): 140 NaCl, 1.2 CaCl_2_, 3.0 KCl and 1.0 MgCl_2_) to a concentration of 300 mM and perfused via the microdialysis probe or diluted in saline (0.9% NaCl) to a concentration of 18% (v/v) and administrated intraperitoneally (i.p.; 2.3 g/kg, 16.7 ml/kg). This was true for all experiments (in vitro and in vivo) performed in this study. Analox enzyme (Analox Instruments, London, UK) was dissolved and diluted with Analox reagent kit (Analox Instruments, London, UK).

### In vivo microdialysis

The animals were deeply anesthetized using isoflurane (Baxter, Kista, Sweden) at 4% during induction before being mounted onto a stereotaxic instrument (David Kopf Instruments, AgnTho’s, Lidingö, Sweden) and placed on a heating pad to prevent hypothermia. The anaesthesia was continued using isoflurane at 2–3% throughout the surgery. A sagittal incision was made, the skull was exposed, and a hole was drilled above the nAc, and two additional holes were drilled for attachment of anchoring screws. A custom made I-shaped probe, with a molecular cut-off of 20 kDa and an active space of 2 mm, was gently lowered into coordinates approximating the nAc core–shell borderline region (A/P: + 1.85, M/L: − 1.4 relative to bregma; D/V: − 7.8 relative to dura mater; Paxinos and Watson 7 th compact ed. 2018). The probe together with the two anchoring screws were fixed to the skull using Harvard cement (DAB Dental AB, Gothenburg, Sweden). Rats were housed individually and allowed to recover for 48 h prior to the in vivo microdialysis experiment. At the day of the in vivo microdialysis experiment, the microdialysis probe was connected to a microperfusion pump (U-864 Syringe Pump, AgnTho’s, Lidingö, Sweden) and perfused with Ringer’s solution at a rate of 2 µl/min, initially for 2 h in order to equilibrate the fluid exchange. Dialysis samples (40 µl) were then collected every 20 min. After obtaining baseline samples, local (300 mM, nAc) or systemic administration of alcohol (2.3 g/kg, i.p.) was administered and another 7 samples were collected. In a final study, female rats received 50 µM taurine alone or concomitantly with alcohol (300 mM) via the dialysis probe, starting at time-point 0 and for the duration of the experiment. Immediately after the experiment, animals were sacrificed and the brains were placed in fixative (Accustain, Sigma-Aldrich, Sweden) after removal. Probe placement was verified after 4–7 days using a vibroslicer (Campden Instruments Ltd, Leicester, UK) and rats with a misplaced probe, local bleeding or other brain tissue damage were excluded from the statistical analysis.

### Biochemical assay

The dialysate samples collected were split and analysed separately with regards to DA and taurine content as previously described (Clarke et al. 2014; Ulenius et al. 2020). In brief, high performance liquid chromatography (HPLC) connected to either an electrochemical detector (for DA), or a fluorescent detector (for taurine), was used for separation and detection of the neurochemicals in the samples. For identification and quantification of the peaks, one external standard containing 3.25 nM of DA and two external standards containing 500 mM and 1000 mM of taurine were used. Sodium azide 20 mM was added to each taurine sample (50% v/v) to maintain stability of the sample. All chromatograms were analysed using Thermo Scientific Chromeleon Chromatography Data System (CDS) software (CHROMELEON7).

### Dialysate alcohol concentration determination

**Fig. 1:**
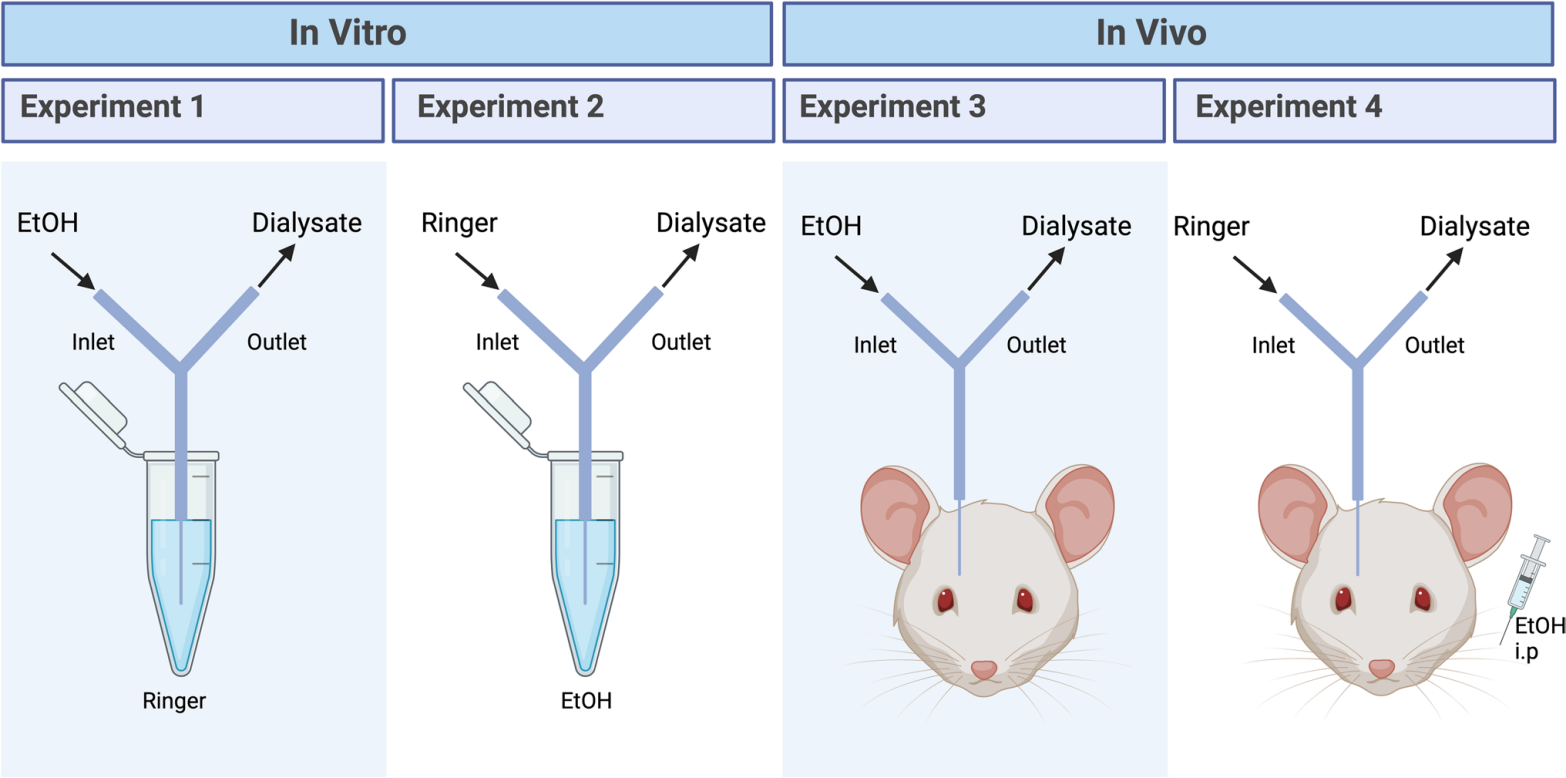
Overview of the experimental design for determination of alcohol concentration. Two separate experiments in vitro were carried out. Experiment 1 mimics local alcohol administration as alcohol (300 mM) was perfused through the probe, whilst Experiment 2 simulates systemic administration with alcohol (50 mM) in the test tube. In Experiment 3, alcohol (300 mM) was given locally in vivo. In Experiment 4, alcohol was given systemically (2.3 g/kg) via i.p. injection in vivo. EtOH alcohol, i.p. intraperitoneal. Figure created using BioRender.

In order to assess if the administered alcohol dose using either local (300 mM, nAc) or systemic (2.3 g/kg, i.p.) drug application using in vivo microdialysis yields similar alcohol concentrations in the brain tissue surrounding the dialysis probe, we conducted a set of in vitro and in vivo experiments measuring the alcohol concentration with an Analox AM1 Alcohol Analyzer (Analox Instruments, London, UK) (Fig. 1). The design of the two in vitro studies was simulating the setup of in vivo microdialysis experiments utilizing either local or systemic alcohol administration. In the first in vitro experiment (Experiment 1) a dialysis probe was placed in Ringer’s solution (1 ml) and perfused with alcohol (300 mM) and the alcohol concentration in the dialysate was analysed. This to simulate reverse in vivo microdialysis and calculate how much of the perfused alcohol diffuses over the dialysis membrane out to the surrounding environment (excovery). In the second experiment (Experiment 2) Ringer’s solution was perfused through the dialysis probe placed in an alcohol (50 mM) solution, simulating in vivo microdialysis with systemic alcohol administration. The concentration of 50 mM used was based on an estimated alcohol concentration in the brain following systemic administration. In this experiment, the aim was to calculate the amount (in percent) of alcohol passing over the membrane into the probe (recovery) by measuring the alcohol concentration obtained through the probe after passing the alcohol-containing solution. The recovery rate was used to calculate the tissue concentration of alcohol following alcohol administration in vivo in Experiment 4 (see below). In the corresponding in vivo studies, the dialysate alcohol concentration was measured in a set of rats with a microdialysis probe implanted into the nAc, receiving alcohol locally (Experiment 3) or systemically (Experiment 4) (300 mM, nAc and 2.3 g/kg, i.p., respectively). In Experiment 3, male rats (n = 3) received local alcohol administration into the nAc. The alcohol concentration in the dialysate was analysed to calculate the amount of alcohol passing over the membrane into the brain (excovery). In Experiment 4, male (n = 6) and female (n = 9) rats received systemic alcohol administration and the amount of alcohol at the outlet of the dialysis probe was analysed. The alcohol concentration in the adjacent brain tissue was calculated based on the recovery rate obtained from the corresponding in vitro experiment (Experiment 2). A flow rate of 2 μl/min was used for both the in vivo and in vitro experiments and samples were collected every 20 min (5–9 samples from each probe/animal depending on the experimental setup). From these samples, 10 μl were diluted (1:10) in Ringer’s solution, before the diluted sample (10 μl) was analysed using the Analox. The samples were run in triplicate and the results were averaged to obtain a final value.

### Statistics

Data was statistically evaluated using GraphPad Prism 10 Software (San Diego, CA, USA). Two-way analysis of variance (ANOVA) with repeated measures (treatment group x time) was used for statistical evaluation of data over time (*t* = 20–140 min) and unpaired *t*-tests were used for statistical analysis of baseline DA and taurine levels as well as for area under the curve (AUC). All data are expressed as means ± standard error of the mean (SEM) and statistical significance considered as probability value (*p*) less than 0.05.

## Results

### Systemic administration of alcohol induces increased extracellular levels of accumbal DA in both females and males without any sex difference

**Fig. 2:**
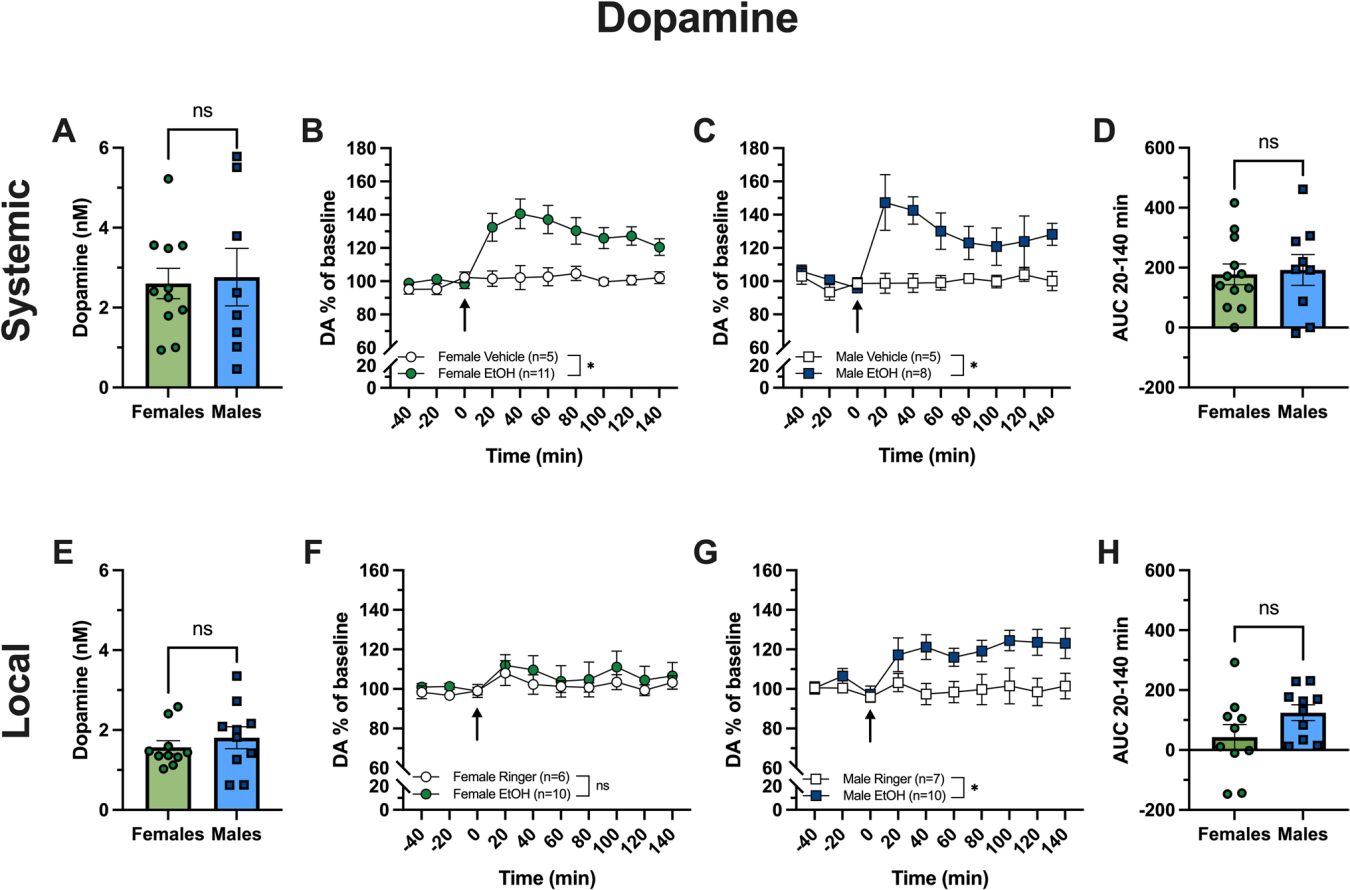
Effect of systemic (2.3 g/kg i.p.) and local (300 mM) alcohol administration on extracellular dopamine levels in male and female rats. Baseline levels of dopamine (DA) (nM) in the dialysate were similar in female and male rats prior to systemic (**A**) and local (**E**) alcohol administration. Time-course graphs showing % of baseline DA (**B**–**C**, **F**–**G**). Arrows indicate injection of alcohol i.p. (**B**–**C**) or start of alcohol perfusion into the nucleus accumbens (**F**–**G**) at timepoint 0. Systemic alcohol administration increased extracellular levels of DA in both female (**B**) and male (**C**) rats. No significant increase in extracellular DA was seen following local alcohol administration in females (**F**), whilst an increase was observed in males (**G**). The area under the curve (AUC) for timepoints 20–140 min between males and females was compared (**D**, **H**). No sex difference in DA change was observed following neither systemic (**D**) nor local (**H**) administration of alcohol. Data are presented as mean values ± SEM. *EtOH* alcohol, *n* number of animals, *ns* non-significant

In the first set of experiments, extracellular levels of DA were monitored following systemic administration of alcohol (2.3 g/kg, i.p.). Baseline nAc levels of DA in female and male rats did not differ (unpaired *t*-test of baseline levels, Females vs Males; t_(17)_ = 0.214, *p* = 0.833; Fig. 2A), and alcohol elevated extracellular DA in both females (two-way ANOVA over timepoints 20–140 min, Veh vs EtOH, group effect; F_(1,14)_ = 10.7, *p* = 0.006; Fig. 2B) and males (two-way ANOVA over timepoints 20–140 min, Veh vs EtOH, group effect; F_(1,11)_ = 6.948, *p* = 0.023; Fig. 2C). No significant difference in the observed alcohol-induced DA increase could be seen between females and males (unpaired *t*-test of AUC over timepoints 20–140 min, Females vs Males; t_(19)_ = 0.248, *p* = 0.807; Fig. 2D).

### Local administration of alcohol into the nAc increases extracellular levels of DA in males but not female rats

Since accumbal mechanisms are important for alcohol-induced DA release, we next outlined putative sex-dependency following local alcohol administration. Pre-drug levels of accumbal DA did not differ significantly between female and male rats (unpaired *t*-test of baseline levels, Females vs Males; t_(18)_ = 0.757, *p* = 0.459; Fig. 2E). Local administration of alcohol (300 mM) into the nAc did not significantly increase extracellular levels of DA in female rats (two-way ANOVA over timepoints 20–140 min, Ringer vs EtOH, group effect; F_(1,14)_ = 0.289, *p* = 0.599; Fig. 2F). However, when administering alcohol (300 mM) locally into the nAc of male rats, a significant increase of extracellular DA was seen as compared to vehicle treatment (two-way ANOVA over timepoints 20–140 min, Ringer vs EtOH, group effect; F_(1,15)_ = 7.816, *p* = 0.014; Fig. 2G). However, no significant difference in alcohol-induced DA increase was demonstrated between females and males (unpaired *t*-test of AUC over timepoints 20–140 min, Females vs Males; t_(18)_ = 1.642, *p* = 0.118; Fig. 2H).

### Extracellular taurine levels in the nAc are increased following systemic administration of alcohol in both females and males

**Fig. 3:**
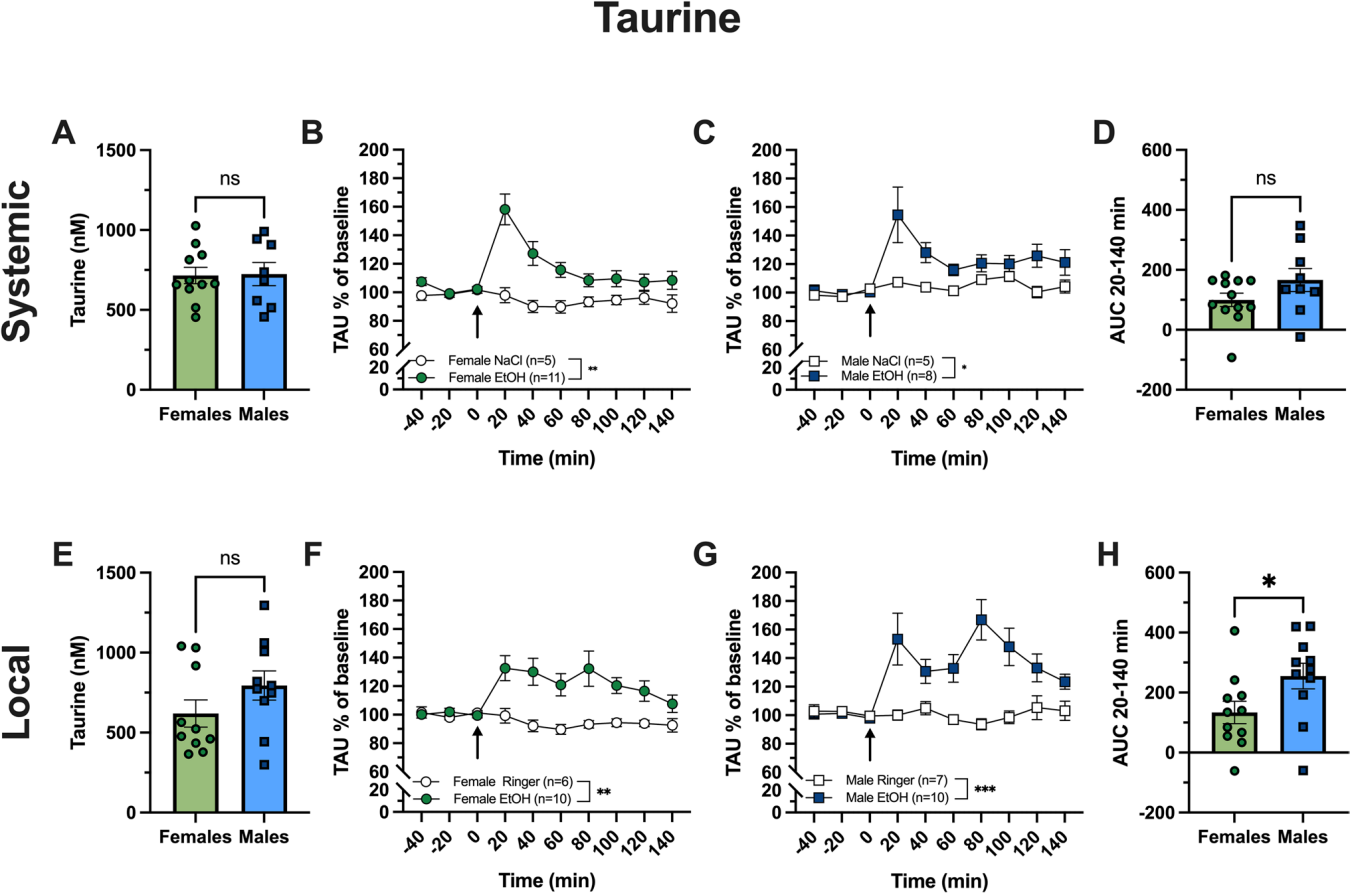
Effect of systemic (2.3 g/kg i.p.) and local (300 mM) alcohol administration on extracellular taurine levels in male and female rats. Baseline levels of taurine (TAU) (nM) in the dialysate (**A**, **E**) were similar between females and males prior to systemic (**A**) and local (**E**) alcohol administration. Time-course graphs showing TAU following alcohol administration, presented as % of baseline (**B**, **C**, **F**, **G**). Arrows indicate injection of alcohol i.p. (**B**, **C**) or start of alcohol perfusion into the nucleus accumbens (**F**, **G**). A significant increase in extracellular TAU levels was seen following both systemic and local alcohol administration in females (**B**, **F**) and males (**C**, **G**). The area under the curve (AUC) for timepoints 20–140 min between males and females was compared (**D**, **H**) and the TAU increase induced by systemic alcohol administration did not differ between sexes (**D**). However, the TAU increase induced by local alcohol administration was significantly higher in male than female rats (**H**). Data are presented as mean values ± SEM. *EtOH* alcohol, *n* number of animals, *ns* non-significant

The raise of extracellular taurine is suggested to be a prerequisite for alcohol-induced DA release. Basal levels of accumbal taurine did not differ significantly between males and females (unpaired *t*-test of baseline levels, Females vs Males; t_(17)_ = 1.05, *p* = 0.918; Fig. 3A). Systemic alcohol administration (2.3 g/kg, i.p.) increased accumbal taurine in both sexes (females: two-way ANOVA over timepoints 20–140 min, Veh vs EtOH, group effect; F_(1,14)_ = 13.60, *p* = 0.002; Fig. 3B; and males: two-way ANOVA over timepoints 20–140 min, Veh vs EtOH, group effect; F_(1,11)_ = 5.041, *p* = 0.046; Fig. 3C). When comparing the alcohol-induced taurine elevation between the sexes, no significant difference was seen (unpaired *t*-test of AUC over timepoints 20–140 min, Females vs Males; t_(19)_ = 1.58, *p* = 0.131; Fig. 3D).

### Alcohol locally perfused into the nAc increases extracellular taurine levels in both females and males

Accumbal taurine levels were monitored during local alcohol (300 mM) administration into the nAc. Baseline levels of taurine in the nAc prior to local alcohol administration did not differ significantly between sexes (unpaired *t*-test of baseline levels, Female vs Male; t_(18)_ = 1.40, *p* = 0.178; Fig. 3E). Local alcohol administration increased extracellular levels of taurine in both female (two-way ANOVA over timepoints 20–140 min, Ringer vs EtOH, group effect; F_(1,14)_ = 10.59, *p* = 0.006; Fig. 3F) and male rats (two-way ANOVA over timepoints 20–140 min, Ringer vs EtOH, group effect; F_(1,15)_ = 18.26, *p* < 0.001; Fig. 3G). However, the alcohol-induced taurine elevation was significantly higher in males compared to females (unpaired *t* test of AUC over timepoints 20–140 min, Females vs Males; t_(20)_ = 2.139, *p* < 0.045; Fig. 3H).

### Exogenous taurine does not alter dopamine response in female rats following local alcohol administration

**Fig. 4:**
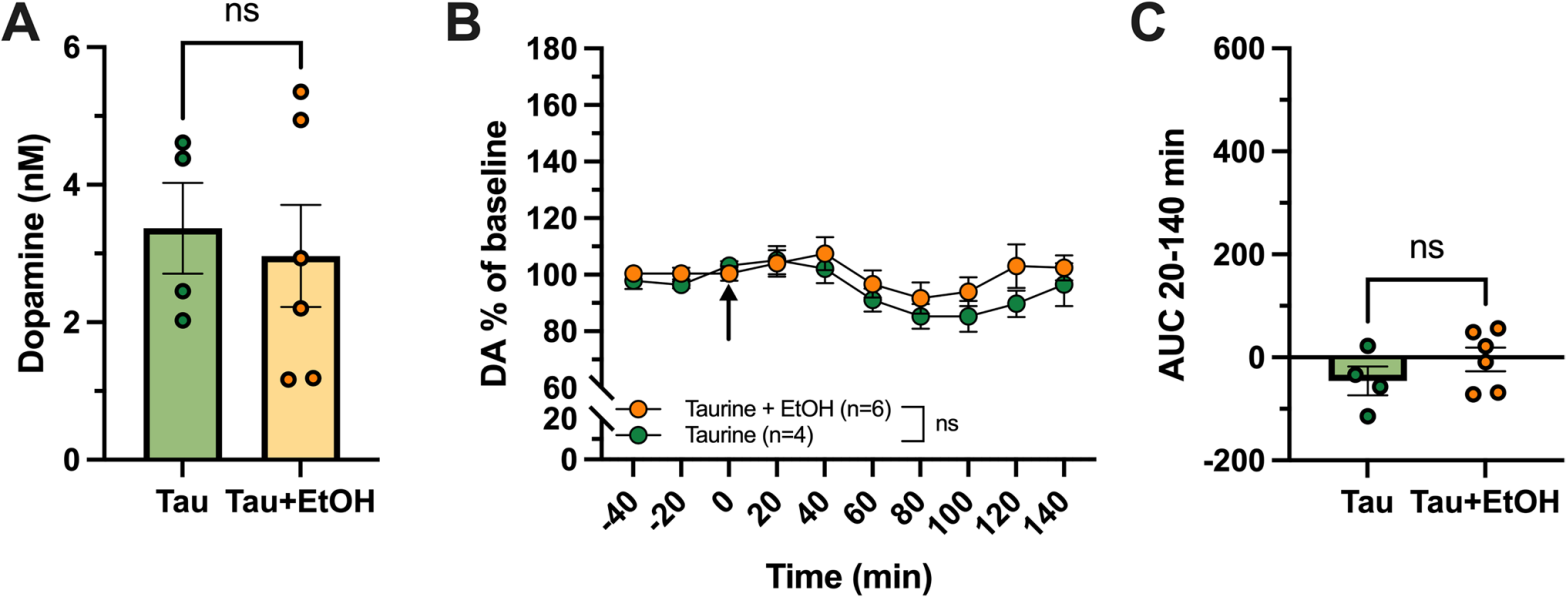
Co-perfusion of taurine and alcohol into the nucleus accumbens of female rats does not induce a dopamine increase. Baseline levels of dopamine (DA) (nM) in the dialysate did not differ between treatment groups (**A**). Time-course graph showing DA % of baseline, drug perfusion started at time-point 0 as indicated by the arrow (**B**). Area under the curve (AUC) over timepoints 20–140 min (**C**). No difference in extracellular DA levels was seen following local perfusion of alcohol together with taurine compared to controls only receiving taurine perfusion (**B**, **C**). *Tau* taurine, *EtOH* alcohol, *n* number of animals, *ns* non-significant

No significant increase in extracellular DA was observed following local alcohol administration in female rats (Fig. 2F). At the same time, a relatively blunted release of taurine was detected in females compared to males (Fig. 3H). To investigate whether the absence of DA response in female rats could be attributed to their lower taurine elevation, further experiments were performed. These involved simultaneous administration of exogenous taurine and local alcohol into the nAc. Baseline DA levels did not differ between treatment groups (unpaired *t*-test of baseline levels, Taurine vs Taurine + EtOH; t_(8)_ = 0.380, *p* = 0.714; Fig. 4A). When co-administering taurine (50 μM) and alcohol (300 mM), no difference in DA levels were observed, compared to taurine alone (two-way ANOVA over timepoints 20–140 min, Taurine vs Taurine + EtOH, treatment effect; F_(1,8)_ = 1.213, *p* < 0.303; Fig. 4B; unpaired *t* test of AUC over timepoints t20-140, Taurine vs Taurine + EtOH; t_(8)_ = 1.153, *p* = 0.282; Fig. 4C).

### Alcohol concentration in brain tissue

**Fig. 5:**
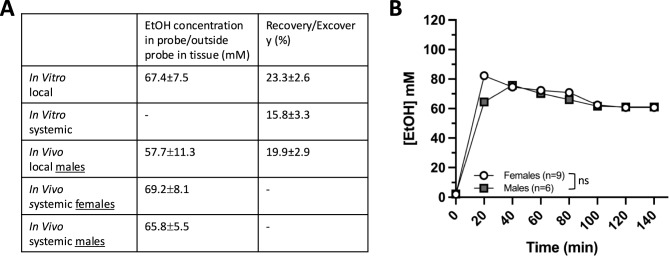
Alcohol concentration over time in female and male nucleus accumbens following systemic administration. Table presenting alcohol concentration in brain tissue and the recovery/excovery rates (**A**). Time-course graph showing the alcohol concentration (mM) in the nAc of females and males following systemic administration (**B**). *EtOH* alcohol, *n* number of animals, *ns* non-significant

To estimate the alcohol concentration reaching the brain tissue following local and systemic alcohol administration (300 mM; 2.3 g/kg i.p.), in vitro experiments simulating in vivo setups were conducted. In Experiment 1, alcohol (300 mM) was perfused through the dialysis probe. To assess how much alcohol was lost in the tubing, alcohol concentration was measured before the start of perfusion and again after passing the tubing material without a connecting probe. In the experimental setup used in Experiment 1, 15.05 mM was lost. After correcting for alcohol lost in the tubing, 67.4 ± 7.5 mM was passing over the membrane of the dialysis probe. This corresponds to an excovery of 23.3 ± 2.6%. For Experiment 2, Ringer’s solution was perfused through the probe placed in a solution containing 50 mM alcohol. The concentration of alcohol in the dialysate was 7.89 ± 1.6 mM corresponding to a recovery rate of 15.8 ± 3.3% (Fig. 5A). In this experimental setup, the dialysate was collected directly at the outlet of the probe, hence the loss of alcohol in tubing was considered negligible. We next conducted in vivo experiments. In Experiment 3, alcohol (300 mM) was perfused through the dialysis probe implanted into the nAc of male rats. In this experimental setup where the probe was connected to an animal, a longer tubing was used between the inlet and the outlet where the dialysate was collected. The alcohol lost in the tubing material was here 30.1 mM. After correcting for alcohol lost in the tubing, 57.7 ± 11.3 mM alcohol was passing through the membrane into the surrounding brain tissue. This corresponds to an excovery of 19.9 ± 2.9% when comparing to the calculated amount of alcohol in the perfused solution at the membrane (adjusted for a loss of 15.05 mM from inlet to probe). In Experiment 4, alcohol was administered systemically (2.3 g/kg, i.p.) to male and female rats. The alcohol concentration reaching the dialysate was 9.9 ± 0.8 mM for males and 10.4 ± 1.2 mM for female rats. The loss in tubing in the in vitro experiments was five percent. Adjusting for loss in tubing, the estimated amount of alcohol passing over the membrane from the brain into the probe was 10.4 ± 0.9 mM for male and 10.9 ± 1.3 for female rats. By using the recovery rate calculated from Experiment 2 (15.8%) the alcohol concentration in the close proximity to the dialysis probe was estimated to 65.8 ± 5.5 mM for males and 69.2 ± 8.1 mM for females. The amount of alcohol reaching the brain tissue surrounding the probe did not differ significantly between sexes (unpaired t-test t_(12)_ = 0.901, *p* = 0.385; Fig. 5A, B).

## Discussion

Excessive alcohol consumption causes medical consequences in both men and women, with known sex differences in the disease progress and the aetiology (Bizzaro et al. 2023; Kirsch et al. 2025). Despite this, clinical and preclinical alcohol research have traditionally predominantly been carried out in male subjects. The alcohol-induced increase of accumbal DA and taurine is a well-known phenomenon in male rodents (Ademar et al. 2024; Dahchour et al. 1996; De Witte et al. 1994; Ericson et al. 2011, 2020), whilst comparative studies using males and females are scarce. This study aimed to investigate if there are any sex differences in DA or taurine signalling following alcohol administration, possibly explaining part of the behavioural differences seen in e.g. alcohol consumption. By means of in vivo microdialysis, we show that there is no sex difference in alcohol-induced increase of DA or taurine following systemic administration in Wistar rat. However, during local administration in the nAc, a significant DA elevation was observed in males only, and the relative taurine release was significantly higher in males compared to females. As we further demonstrate that equal alcohol concentrations in the brain tissue are observed following different administration routes, we conclude that alcohol may affect accumbal mechanisms in a partially sex-specific manner.

Female rodents display a higher voluntary alcohol intake than their male counterparts in various alcohol consumption paradigms (Rath et al. 2020; Sneddon et al. 2019). The rewarding effect of alcohol is mediated via complex mechanisms but increased extracellular levels of DA within the nAc appear to be central. Increased accumbal DA has repeatedly been shown following both local and systemic alcohol administration in rodents (Di Chiara and Imperato 1988; Ericson et al. 2020; Imperato and Di Chiara 1986; Loftén et al. 2021) and indications of increased striatal DA following alcohol administration have also been demonstrated in humans (Boileau et al. 2003). A higher striatal DA increase has been shown in men than in women following oral alcohol administration in non-alcoholic study subjects (Urban et al. 2010). Whether possible sex differences in alcohol-induced DA response in rodents contribute to the difference in consummatory behavior, is not clear. A previous study, using Long-Evan rats, showed a larger alcohol-induced DA release within the nAc in female rats than males following systemic alcohol administration (Blanchard and Glick 1995). In the present study, we show a significant increase in accumbal DA following systemic administration of alcohol in both female and male Wistar rats without any sex difference (Fig. 2B–D), highlighting possible strain differences. In contrast to the human imaging study reporting a sex difference in alcohol-induced DA elevations (Boileau et al. 2003), alcohol was here administered via an i.p. injection and thus by-passed the intragastric alcohol metabolism that is known to differ between sexes and may contribute to the different results obtained in the Wistar rat.

Alcohol produces its pharmacological effects via several receptor types and neurocircuitries in an intricate manner (Abrahao et al. 2017). To study the effect of alcohol in specific neurocircuitries with the aim of detangle the mechanisms behind its DA releasing and reinforcing effects, local application of the substance can be utilized. When applying alcohol locally into the nAc, a significant increase in extracellular levels of DA was observed in male rats but not in females. However, no significant difference in alcohol-induced DA levels could be detected between sexes (Fig. 2H). When investigating the pre-drug baseline levels of DA, there were no differences between the female and male rats (Fig. 2A, E). The DA response in female rats following the local alcohol administration was characterized by a larger intra-group variance than what was obtained in male rats, which partly could explain the non-significant change in DA levels on a group level. The variability in DA response to alcohol in females could possibly be explained by cyclic variations as it has been shown that VTA DA neurons fire differently depending on the current stage of the oestrous cycle and that oestradiol stimulates DA release (Becker et al. 2012). Also, basal extracellular DA levels of dorsolateral striatum have been demonstrated to fluctuate across the oestrous cycle (Xiao and Becker 1994). In this study, the stage of the oestrous cycle was not determined at the day of the experimental procedure and thus was not accounted for. Worth noting is that the firing pattern of DA neurons are influenced by sex hormones in both females and males, but with larger fluctuations in females along the oestrous cycle (Becker et al. 2012). It is also possible that other components of the mesolimbic DA system than the nAc play a larger role in the alcohol-induced DA increase in female rats, e.g. activation within the VTA, as the response to systemic administration was similar. The contribution of different neuronal components is not possible to determine with the experimental set-up used in this study.

Alcohol-induced DA elevation in the nAc is a robust, but complex, phenomenon involving a cascade of neurochemical events. In male rats, the increase in DA has been shown to be preceded by a required rise in extracellular taurine within the nAc (Adermark et al. 2011; Ericson et al. 2011), a phenomenon that has so far not been verified in the female counterpart. In this study, we show that both systemic and local administration of alcohol results in significant increases in extracellular levels of accumbal taurine in male rats. To our knowledge, this is the first study that presents alcohol-induced taurine elevation within the nAc, both through systemic and local drug administration, also in female rats. When examining the basal levels before ethanol treatment, females and males were found with extracellular taurine levels of the same magnitude (Fig. 3A, E). Following systemic administration, no significant difference in the alcohol-induced taurine levels between sexes was found (Fig. 3D) whilst a significantly higher taurine increase was noted following local alcohol application in male rats as compared to females (Fig. 4H). Concurrently, the females displayed a blunted DA response following local alcohol administration. To assess if the blunted DA response was due to a lower level of alcohol-induced taurine release, taurine was applied concomitantly with alcohol into the nAc. In a previous study using male rats, this relatively low concentration of taurine was sufficient to restore alcohol’s ability to elevate DA when the alcohol-induced taurine increase was blocked (Ericson et al. 2011). However, no additional effect on DA was observed in the female rats (Fig. 4B, C), suggesting that other mechanisms may explain the blunted DA response.

Outbred Wistar rats were used in this study in an attempt to mirror the genetic heterogeneity seen in the human population. However, a great variability in alcohol-related behaviours has been shown between Wistar rats from different vendors (Palm et al. 2011) possibly in part due to genetic variations, meaning that this probably also holds for different batches from the same vendor. Studies have presented the presence of genetic variability in alcohol-induced taurine release within the nAc. Selectively bred high-alcohol sensitive (HAS) and low-alcohol sensitive (LAS) rats were found to differ in their neurochemical profile to alcohol (2.0 g/kg, i.p.) when HAS rats responded with a transient elevation of extracellular taurine in the nAc, while the onset of the alcohol-induced taurine increase in the LAS rats was delayed two hours following the injection (Dahchour et al. 2000). Also, when investigating mice lacking the epsilon form of protein kinase C (PKCε), which has been shown specifically sensitivity to alcohol, no alcohol-stimulated increase in mesolimbic taurine nor DA levels were observed (Olive et al. 2000). Thus, genetic variations are an important factor impacting experimental outcomes and need to be considered. Even if the experiments in this study to a high extent were carried out in parallel, batch-related effects might contribute to variability.

In the present study, the systemically administrated dose of alcohol used was 2.3 g/kg and the alcohol concentration locally perfused into the nAc was 300 mM. Previous studies using similar concentrations of alcohol (2.5 g/kg i.p. (Blomqvist et al. 1997); 300 mM, nAc (Ericson et al. 2003)) have found a comparable increase in accumbal DA levels, with an elevation of about 30%. In this study, we aimed to determine if systemic administration (2.3 g/kg, i.p.) and local perfusion (300 mM) of alcohol yield equivalent concentrations of alcohol in the tissue surrounding the dialysis probe. We found that in vitro local alcohol administration resulted in an alcohol concentration of 67.4 mM near the probe, while the corresponding in vivo experiment yielded an alcohol concentration of 57.7 mM. The excovery rates for these conditions were 23.3% and 19.9%, respectively (Fig. 5A). A slightly lower excovery was observed in the in vivo experiments, which could be attributed to potential clogging or membrane damage caused by the implantation of the probe into the rat brain. This could explain the small but existing difference in excovery rates. To assess the alcohol concentration near the probe after systemic alcohol administration, an in vitro experiment was conducted to determine the recovery rate in the experimental setup used. The recovery rate was 15.8%, which was assumed to be applicable to the in vivo setting and used to calculate the concentration of alcohol in the surrounding tissue of the implanted probe. The concentrations were 69.2 mM for females and 65.8 mM for males. These concentrations are highly similar to the in vitro local alcohol concentration of 67.4 mM and are relatively close to the in vivo local administration of 57.7 mM. Both routes of administration resulted in comparable concentrations of alcohol within the target tissue. However, the recovery and excovery rates differed slightly (15.8 and 19.9–22.1, respectively). This difference could be due to the lower concentration gradient in the recovery conditions, with 50 mM in the in vitro studies and estimated 65.8–69.2 mM in the in vivo setting, compared to the 300 mM alcohol solution used for local administration. Additionally, we confidently estimate that a similar concentration of alcohol reaches the target brain area after its systemic administration (i.p.) of both male and female rats (Fig. 5B). Depending on the research question, both routes of drug administration can be valuable in preclinical studies. Systemic administration has a higher clinical relevance, whilst local perfusion of alcohol is useful for local mechanistic studies in specific target areas.

Studies investigating possible sex differences in alcohol pharmacology, including activation of the mesolimbic DA system, are important, since tentative differences observed in the extension could inform the development of equally effective AUD treatment options for men and women. In this study, we show that male and female rats display a similar DA and taurine response following systemic administration of alcohol. In contrast, males display a significant increase in both DA and taurine following intra-accumbal administration of alcohol whilst female rats display a blunted DA response and an attenuated taurine increase, suggesting that local accumbal mechanisms may be more involved in male than in female rats.

## Data Availability

Data are available from the corresponding author upon reasonable request.
